# Automated assessment of infant motor development to predict infant age: The determination of objective metrics of spontaneous kicking

**DOI:** 10.1017/wtc.2022.25

**Published:** 2022-11-23

**Authors:** Katelyn Fry-Hilderbrand, Yu-Ping Chen, Ayanna Howard

**Affiliations:** 1 Institute for Robotics and Intelligent Machines, Georgia Institute of Technology, 85 5th St. NW, Atlanta, GA 30308, USA; 2 Department of Physical Therapy, Georgia State University, 140 Decatur St., Atlanta, GA 30303, USA; 3 College of Engineering, The Ohio State University, 2070 Neil Ave., Columbus, OH 43210, USA

**Keywords:** Sensors, Performance characterisation, Rehabilitation robotics

## Abstract

Though early intervention can improve outcomes for children with motor disabilities, delays in diagnosis can impact the success of intervention programs. Prior work indicates that spontaneous kicking patterns can be used to model typical infant motor development to assist in the early detection of motor delays. However, abnormalities in spontaneous movements are not well defined or readily observable through traditional functional assessments. In this research, a method is introduced for the early detection of delays through the assessment of spontaneous kicking data gathered using a wearable sensing suit. We present formulations of kinematic features identified in the clinical space, identify which features are significant predictors of infant age, and establish normative values. Finally, we offer an analysis of preterm (PT) infant data compared to normative values derived from term infants. Term and PT infants ranging in age from 1 to 10 months were studied. We found that frequency, duration, acceleration, inter-joint coordination, and maximum joint excursion metrics had a significant correlation with age. From these features, models of typical kicking development were created using data from term, typically developing infants. When compared to normative trends, PT infants display differing developmental trends.

## Introduction

According to the US Department of Health and Human Services and the Center for Disease Control and Prevention, 10.09 % of all births in the United States are considered premature, where the gestation period of the infant is 37 weeks or less (Hamilton et al., 2021). In the United Kingdom, it is estimated that 7.9 % of births are premature while worldwide it is estimated that about 15 million births, or between 9 and 12 %, are premature (Chawanpaiboon et al., 2019). In addition, a study by the World Health Organization suggests that these rates of premature births are increasing in many countries (World Health Organization, 2015). Premature birth is a leading cause of infant morbidity and mortality (Lee et al., 2019; Dahman, 2020). According to the US National Institute of Health, the leading cause of infant death is a preterm (PT) birth (NICHD, 2017). As such, there have been many advances and improvements made by the medical field in neonatal care with a focus on reducing infant mortality rate, such that the survival rate of an infant born 4 weeks early was less than 60 % in 1980 but increased to over 80 % by the mid-2000 (Glass et al., 2015). In conjunction with the increase in survivability of premature birth infants, there is an increase in morbidity. Indeed, premature births are associated with approximately 50 % of all disabilities in children (Glass et al., 2015). Such morbidities include many neurodevelopmental abnormalities and delays in reaching motor skill milestones.

Delays in reaching early motor skills interfere with a child’s ability to coordinate muscle groups. Infants who are delayed in reaching early motor skill milestones tend to have difficulty with more advanced milestones, usually resulting in delayed walking onset and fine motor delays (Grow, 2020). Early detection of neurodevelopmental abnormalities, allowing for early intervention practices, has been shown to reduce the associated symptoms and disabilities, thereby leading to an improvement of mobility and overall quality of life. Studies have shown treatments and therapies may improve the overall quality of life of affected individuals if developmental delays can be reliably detected early in life (Rogers et al., 2015; Subramanyam et al., 2015). It is during the early childhood years that children develop language and communication skills, develop connections to parents and siblings, and learn how to interact with the outside environment. As such, learning how to deal with any limitations is critical during these early years. However, due to the variability between individual cases, it is difficult to design a diagnostic test to encompass all patients (Bryant et al., 2017).

Currently, detecting delays and potential abnormalities in motor development in infancy requires clinical observation and documentation of functional motor milestones in combination with neurological assessments over several months and in some cases years (Hadders-Algra, 2014). These approaches are typically confined to a clinical setting which limits the amount of time an infant can be observed (Smith et al., 2015). Moreover, these approaches are subjective by nature due to their dependence on infant cooperation during the observation time and opinion of the clinician. Given these limitations, the development of objective tools to improve early detection, outside of direct clinical observation, is needed.

In this paper, we introduce a method for the early detection of possible delays through the assessment of infant motor development using inertial sensors placed on an infant’s lower limb segments. We introduce formulations of kinematic features identified in the clinical space. We then identify which of these features are significant predictors of infant age and establish normative values at various ages. Finally, we offer an analysis of PT infant data compared to normative values derived from term infants.

## Related works

Rather than delays in the attainment of voluntary motor skills, clinicians oftentimes observe abnormalities in the quality of an infant’s spontaneous movements. Prior work indicates that infant spontaneous kicking patterns can be used to assist in the modeling of typical infant motor development. Spontaneous kicking is one of the earliest displays of coordinated motor skills (Kanemaru et al., 2012). This self-initiated motor behavior involves movements of various body parts with a fluent and complex appearance (Jeng et al., 2004; Fetters et al., 2010). Additionally, spontaneous kicking is directly related to elements of skilled coordination that are seen at later stages of life (Thelen et al., 1987; Jeng et al., 2002). The spatial and temporal patterns of early kicking movements were found to be similar to those involved in mature walking cycles and kicking may be a developmental manifestation of a central program later used for locomotion (Thelen and Fisher, 1983; Fetters et al., 2010). Therefore, spontaneous kicking is an important indicator of later motor development.

However, abnormalities in spontaneous movements are not well defined and are not readily observable through traditional functional motor milestone assessments. To this end, several features of spontaneous kicking have been studied and identified in the clinical space to distinguish between typical and atypical motor development. For example, Jeng et al. (2002) find that the kick frequency, the number of kicks by each leg per minute, increases with age for infants with very low birth-weight and low gestational age. Additionally, Smith et al. (2015) find that infants tend to be less active in their kicking as they approach crawling and walking age indicated by a decrease in kick frequency. A “coordination pattern” is a period of bilateral or unilateral kicking. Bilateral kicking is a period of activity where the infant is moving both of their legs and can include synchronous, reciprocal (asynchronous), or other bilateral coordination patterns. Unilateral kicking is a period of activity where the infant is moving only one of their legs. The frequency of various coordination patterns is called the inter-limb coordination. Clinicians have found that infants typically display more instances of unilateral kicking than synchronous kicking early in life. As they age, there is an observed decreasing percentage of unilateral kicks and increasing percentage of bilateral, synchronous kicking (Jeng et al., 2002; Kanemaru et al., 2012; Trujillo-Priego and Smith, 2017; Chen et al., 2021).

Duration of movement, also called temporal duration, is defined as the duration of time that has elapsed between each pause or change of direction in an infant’s kicking. The usefulness of this metric with respect to age is undetermined as some works find no correlation between average duration and age (Trujillo-Priego and Smith, 2017) while others find an increase with age (Rademacher et al., 2008). A “kick cycle” is defined as a sequence of four distinct phases: flexion of the hip, an intra-kick pause, an extension of the hip, and an inter-kick pause. Fetters et al. (2004) find a difference in the spatiotemporal organization or the duration of each kick phase within a kick cycle. As infants age, the duration of the intra-kick pause increases while the duration of the inter-kick pause decreases. This indicates that the infant spends more time with their leg extended against gravity and spends less time resting between subsequent kick cycles. A “constrained movement” is indicated by invariant durations in the flexion and extension phases of a kick cycle. Clinicians have found that very young infants and older infants with abnormal motor development display constrained movements during a clinically defined kick (Thelen and Fisher, 1983; Einspieler and Prechtl, 2005). As such, this is typically held as a very important metric to detect abnormalities in an infant’s motor development. Various works have defined this metric as the variance in the duration of the flexion and extension movement for each joint within the movement. To our knowledge, no clinical studies have examined the durations of other movements.

Inter-joint coordination can be clinically defined as the pair-wise cross-correlations of joint angles at the hip, knee, and ankle. This metric is used to detect inter-dependencies between joints. These correlations tend to be high during the newborn period and decrease as the infant ages (Jeng et al., 2002; Einspieler and Prechtl, 2005). Additionally, high inter-joint coordination extending into the later months of infancy is held as a good indicator of abnormal motor development (Chen et al., 2021). Jeng et al. (2004) observed a decline in the hip–knee correlation with age but did not note a similar relationship between age and hip–ankle or knee–ankle coordination. This metric is typically calculated during a kick cycle. Joint angle positions and joint angle excursions are popular metrics for describing infant kicking. Jensen et al. (1994), Fetters et al. (2004), and Olivares (2012) compared the joint angle positions at the beginning and end of the flexion and extension phase, the range of motion of hip flexion within each phase (kick amplitude), and the maximum angle positions within each phase between PT and full-term (FT) infants. Fetters et al. (2004) find that FT infants have significantly more flexed hip and knee flexion angles at peak flexion than does the PT group. Additionally, the PT group has significantly smaller hip joint excursions during the flexion phase of the leg movement in comparison to the FT group. Significance of additional metrics in differentiating between the PT and FT group is not found or agreed upon between studies. These studies, however, did not offer analysis of these metrics with respect to age. Jeng et al. (2004) offer an analysis of kick amplitude with respect to age. They found that infants exhibit a larger kick amplitude with increasing age.

Finally, acceleration of the foot is examined in multiple works as a possible feature that correlates with infant age or can help indicate potential abnormalities in motor development. Typically, the magnitude of the foot’s acceleration is taken for each movement within a data segment of 1-min length. The peak and average acceleration is then determined for that minute. However, Trujillo-Priego and Smith (2017) find no relationship between either metric or infant age.

## System description and data collection procedure

The system described in this section has been used in previous works to gather motion data associated with an infant’s spontaneous kicking patterns (Fry et al., 2018, 2019). Our system, hereafter known as the baby SmartyPants, is an embedded sensor suit which, when coupled with a custom data collection app, gathers infant kinematic kicking data over long periods of time. This system is designed to be used with minimal training so that parents will be able to confidently deploy the system in their homes. Additionally, a custom app that can be run on devices running Android mobile OS is created to enable data collection by parents.

The data collection procedure described in this section is performed by members of the research team with parental cooperation, though system setup and data collection could feasibly be performed by the parents with minimal training as in Fry-Hilderbrand et al. (2021). The experimental procedures involving human subjects described in this section are approved by the Institutional Review Boards of the Georgia Institute of Technology and Georgia State University. Parents of the infants consented to the experimental procedures.

### Infant sensing suit

**Figure 1.**
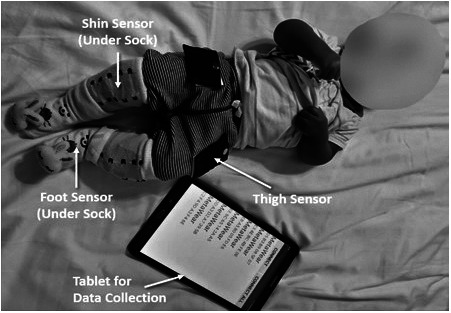
Setup of the SmartyPants system deployed to the home of a preterm, at-risk infant. IMU inertial sensors are placed on each thigh, shin, and foot. Tablet used for data collection also shown.

Our system couples a Bluetooth-connected infant sensor suit with a data collection app, resident on a mobile device to enable ease of collection in the home. The infant sensor suit incorporates six wireless inertial measurement unit (IMU) sensors, placed on each thigh, shin, and foot. The IMU sensors used for the infant suit are MbientLab’s MetawearC wireless sensors (diameter 24 mm, weight 0.2 oz). These lightweight sensors gather 3-axis acceleration and gyroscope data for each of the limb segments and utilize Bluetooth Low Energy technology for data transfer to our custom app. The custom app allows clinicians or parents to gather data from the sensor suit while monitoring battery life and connectivity of each sensor. The suit used for data collection utilizes Velcro pockets to attach the sensors to a pair of infant pants and socks. Each sensor is powered by a coin-cell battery and is placed inside a pocket before being attached to the clothing at the midpoint of the infant’s limb segment. Additionally, a second pair of socks is placed over the foot sensor and shin sensor as needed to hold the sensor pockets in place (Figure 1).

### Data collection

For observation in the home, infants are placed supine on a flat, padded surface while wearing the infant sensor suit. The infant’s legs are momentarily held stationary at the beginning of each kicking session. This step allows for a later calibration to a zero-point time stamp with respect to quantifying the performance of our algorithms. The infant is then encouraged to kick by providing stimulation consisting of verbal gestural cues and presentation of physical play objects. Stimulation is provided until it is determined that the infant needs rest. The determination for rest is determined by the parent or clinician if the infant exhibited any form of agitation or distress.

Each data collection session lasted approximately 1 hr with 20 min of active data collection. During kicking, acceleration and angular rate data are collected at a sampling rate of 100 Hz from the embedded infant suit sensors. Several periods of data are collected with each session yielding up to 20 min of kicking data. Sessions are also filmed to provide clinicians with a visual representation of the infant’s kicking. The individual coin-cell batteries lasted the entirety of the testing session. Additionally, battery levels after testing did not show a significant drop.

To date, we have gathered data from 23 infants, ranging in age from 1 to 10 months, using the SmartyPants system. Of these infants, 15 are considered FT while 8 are considered PT. Some infants are tested multiple times for a total of 49 sessions of data. The sex, gestational age, age(s) when tested, and adjusted age(s) when tested for each infant are reported in Table 1. The adjusted age is reported only for PT infants, calculated by subtracting the number of weeks early an infant is born from the infant’s birth age.

**Table 1.** Demographics of infants from which data were gathered^a^

Infant ID	Sex	Gestational age (weeks)	Ages tested (months)	Adjusted age (months)
B	F	38	3, 4.5	–
C1	F	32*	5	3
C2	M	32*	5	3
D	F	39	2, 5, 8	–
E	F	39	2, 3.5	–
F	F	34*	2, 3, 5.5, 7.5, 8.5	0.5, 1.5, 4, 6, 7
G1	M	33.5*	2	0.5
G2	M	33.5*	2	0.5
H	F	39	1, 5, 8.5	–
I	M	37*	1, 2.5, 5.5, 7	0.5, 2, 5, 6.5
J	M	39.5	2, 4, 4.5	–
K	F	37.5	2, 3.5, 6.5	–
L	M	37	2, 4.5, 6.5	–
M	M	39.5	3, 5.5	–
N	F	36*	3.5, 5	2.5, 4
O	M	38.5	3	–
P	M	40	3, 5.5	–
Q	F	39	1.5	–
R	F	40	3, 4.5	–
S	F	40	3	–
T	M	40	3, 4.5	–
U	F	40	6	–
V	F	28*	6.5, 8.5, 10	3.5, 5.5, 7

a Gestational age (GA) marked with an asterisk (*) indicates a preterm infant

### Data processing

There are a few sources of measurement error that should be accounted for before data analysis. Firstly, the bias of a rate gyroscope is the average output when it is not undergoing any rotation. Similarly, the bias of an accelerometer is the offset of the output signal from the true value (gravity appearing as a bias of 1 g). These biases determined by collecting acceleration and angular rate data while the sensor is at rest and calculating the long-term average output of each signal. Once these biases are known, they are simply subtracted from the output of the gyroscope and accelerometer, respectively, during data collection, yielding bias-corrected measurements. Secondly, the bias-corrected acceleration and angular rate measurements should indicate constant values of zero acceleration (except for the component due to gravity) and angular rate. However, due to mechanical noise, the signals at rest are non-constant. A median filter is used on the IMU gyroscope and accelerometer data to filter out mechanical noise. Finally, gyroscopes are subject to bias instabilities that cause the initial zero reading to drift over time. A zero-velocity update is typically used to account for this drift. During periods of rest, the angular rate output of the gyroscope is reset to zero, eliminating drift accumulating during periods of movement. Here, an activity detection algorithm is used to determine periods of activity and inactivity (Fry, 2018). Once periods of inactivity are determined, the gyroscope data are parsed through a zero-velocity update to eliminate drift.

After correcting for measurement error, each session of data is divided into 1-min segments. Each kinematic feature as introduced in later sections is computed for each 1-min segment. These kinematic features are analyzed for all 1-min segments of term infant data to determine normative values at various ages. For PT infants, the median value of each kinematic feature across all data segments is compared to normative values at their age of testing to determine potential delays in their development.

## Kinematic features

Potential features to describe spontaneous kicking in typically developing infants have been identified in the clinical space. However, the employment of these clinically defined features oftentimes is not obvious or trivial. Additionally, these clinical terms tend to be defined with respect to specific movements. To utilize these features, it is necessary to generalize features that apply to a broader set of infant movements. Additionally, these features need to be functionalized from their clinical definition to a formulation that is implementable and interpretable.

We discuss our process for algorithmically computing these clinically identified features as well as the identification of potential additional features. We use these features to develop a set of kinematic metrics that describes the characteristics of typical spontaneous kicking at different stages of development. Using these metrics, an infant is considered motor developmentally delayed if their spontaneous kicking displays characteristics that are more typical of a younger infant. An analysis of these features and their relationship to infant age is presented.

### Definitions

To allow for the analysis of a wider selection of infant movement data, we define the following terms. A movement sequence refers to a period of activity and is composed of individual movements and coordination patterns. For a given instance of data, the state of the individual joints is described in a phase vector. The phase vector indicates if the individual joints are at rest or moving in a positive or negative direction for a given instance. As such, a movement is indicated by a period of consecutive, identical phase vectors. We define a coordination pattern as a period of either bilateral or unilateral coordination. Bilateral coordination is a period of movement where both legs are moving at the same time while unilateral coordination is a period of movement where only one leg is moving. Unilateral coordination is defined by introducing a unilateral left and unilateral right coordination pattern. Additionally, given that infants tend to favor one leg early in life, we distinguish unilateral coordination with respect to the predominantly active leg of the infant. The predominant leg for an infant is the leg with the highest frequency of movement, or the leg that moves more often. So, in addition to examining trends in unilateral left and unilateral right coordination with respect to age, we select the unilateral movement data for each infant’s predominant leg to examine predominant unilateral coordination trends with respect to age.

We formulate two vectors to describe when movement sequences and when coordination patterns are occurring in a data segment. An activity vector for each leg is created using a gross activity detection algorithm that uses a threshold to determine when periods of activity are occurring (Fry, 2018). These activity vectors are then combined to create an overall gross activity vector. For data segment 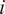 ,
(1)

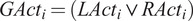

where 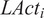 and 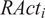 are the activity vectors for the left and right leg, respectively, for data segment 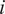 and 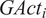 is the overall gross activity vector for data segment 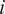 . A value of “0” for a sample in 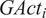 would indicate a sample of no movement. A value of “1” would indicate a sample of activity. A group of consecutive samples of activity in 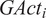 indicate a movement sequence. These activity vectors are also combined to create an overall coordination vector 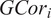 . For data segment 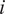 ,
(2)

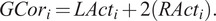

A value of “0” for a sample in 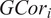 would indicate a sample of no activity (no movement). A value of “1” would indicate a sample of unilateral left activity while a value of “2” would indicate a sample of unilateral right activity. Finally, a value of “3” would indicate a sample of bilateral activity. Groups of consecutive like values in 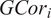 indicate individual coordination patterns.

Finally, we formulate a vector to describe when kick cycles are occurring. According to Fetters et al. (2004), a kick cycle is defined as a period of full flexion followed by a period of full extension. A movement is considered a period of full flexion if both the hip and knee are simultaneously flexing for at least 50 ms, with either the hip or knee joint excursion exceeded 11.5°. A movement is considered a period of full extension if both the hip and knee are simultaneously extending following a period of full flexion. Using this definition, the ankle does not necessarily move in phase with the hip and knee joint during full flexion or full extension. Data associated with periods of full flexion and full extension are stored in 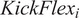 and 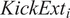 , respectively. Each vector has a length of 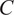 indicating the number of kick cycles within each data segment, 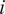 .

#### Frequency of movement

In our implementation, we generalize the kick frequency to measure how often an infant is at rest versus how often they are moving. Additionally, we examine how often the infant performs specific coordination patterns. For the formulas presented, the following variables are used. 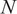 is the total number of samples within a data segment. 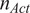 is the number of samples in a data segment that indicate activity for either leg, 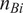 is the number of samples in a data segment that indicate bilateral activity, 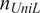 and 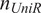 are the number of samples in a data segment that indicate unilateral activity for the left and right leg, respectively, and 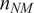 are the number of samples in a data segment that indicate periods of rest.

The *frequency of activity* ( 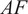 ) is a measure of how long a baby is active within a data segment. This metric is calculated by dividing the number of samples where activity was detected for either leg by the total number of samples in a data segment. For data segment 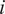 , the frequency of activity is calculated as follows:
(3)

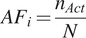



Inter-limb coordination, also called coordination frequency, is a measure of how long a baby performs a specific coordination pattern. This metric is intended to gauge the level of coordination between the left and right leg. For data segment 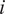 , the coordination frequency for bilateral activity 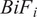 , unilateral left activity 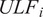 , and unilateral right activity 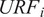 is calculated as follows:
(4)

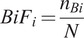



(5)

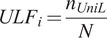



(6)

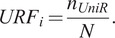



The predominant leg for an infant is the leg with the highest frequency of movement. So, the coordination frequency for predominant unilateral movement, 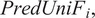 can be calculated by taking the greater of 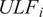 and 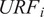 :
(7)

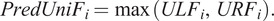



#### Duration of movement

We measure the duration of periods of movement sequences (periods of activity) within a data segment. We then determine the average and maximum duration of movement sequences. Additionally, we measure the duration of bilateral, unilateral left, and unilateral right coordination patterns. For each of these patterns, we determine the average and maximum duration during each data segment.

For the following formulas presented, the following variables are used: 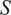 indicates the number of movement sequences as identified by groups of consecutive active values in 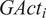 , 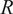 indicates the number of rest sequences as identified by groups of consecutive inactive values in 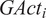 , 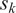 indicates the 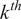 movement sequence where 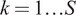 , and 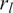 indicates the 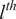 rest sequence where 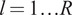 . We also define a vector, kicking duration 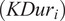 , that stores the duration of each movement as identified by 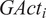 .
(8)

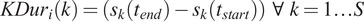

where 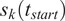 is the start time for the 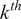 movement sequence and 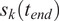 is the end time for the 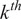 movement sequence. Finally, we define a vector, rest duration 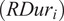 , that stores the duration of each period of rest as identified by 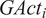 .
(9)

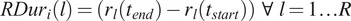

where 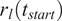 is the start time for the 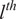 rest sequence and 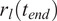 is the end time for the 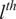 rest sequence. All formulas are presented with respect to a single data segment 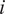 .

The average kicking duration, 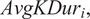 is a metric that determines the mean duration for a period of active kicking and is calculated as:
(10)

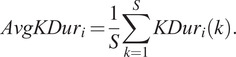

The max kicking duration, 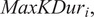 is a metric that determines the maximum duration for a period of active kicking and is calculated as:
(11)

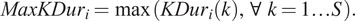

Similarly, the average rest duration, 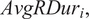 is a metric that determines the mean duration for a period of rest and is calculated as:
(12)

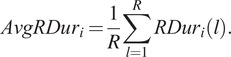

The max rest duration, 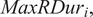 is a metric that determines the maximum duration for a period of rest and is calculated as:
(13)

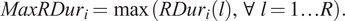



#### Accelerations

We examine the peak and average acceleration of the predominant leg’s foot sensor during movement sequences. The following formulas are calculated for the foot sensor with respect to a single data segment 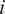 . Additionally, let 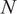 represent the total number of samples within a data segment and 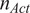 the number of samples in a data segment that indicate activity for the predominant leg.

The average kicking acceleration, 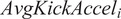 , is the mean magnitude of the acceleration during periods of active movement as identified by 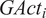 .
(14)

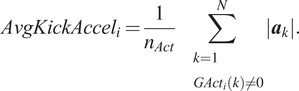

where 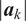 is the magnitude of the 3D vector of acceleration for sample 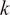 . Note that while the summation occurs over all samples, 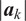 is only included in the sum if 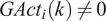 for a given 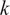 .

The peak kicking acceleration, 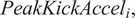 is the maximum magnitude of the acceleration during periods of active movement as identified by 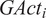 .
(15)

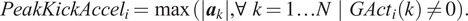

where 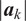 is the magnitude of the 3D vector of acceleration for sample 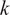 . Note that only samples where 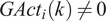 are considered.

#### Maximum joint excursions

We also examine the maximum joint excursion for each degree of freedom (DOF) of the predominant leg and each direction. As stated, the predominant leg for an infant is the leg with the highest frequency of movement. First, the angular rates for each DOF are extracted from gyroscope data of each sensor using a principal component analysis to determine relative joint axes and a decoupling method to isolate each joint’s angular rate data (Fry et al., 2021). Using the angular rates in data segment 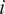 , a vector is then constructed to determine the phase of each DOF, 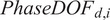 where 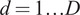 and 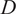 is the total number of DOFs. 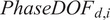 has a length equal to the number of samples in data segment 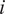 . Periods of positive angular rate values are indicated with a “1” while periods of negative angular rate values are indicated with a “-1.” Periods where resultant joint angle change is less than 5° for 1 s of movement are considered periods of rest, “0.” For a DOF, consecutive samples of positive angular rate values indicate a period of positive phase and consecutive samples of negative angular rate values indicate a period of negative phase.

The joint excursion for each DOF is taken during each period of positive phase for each period of negative phase. The DOF is considered to be at 0° at the beginning of each movement. These joint excursions are stored in 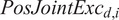 and 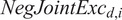 , respectively, for each DOF, 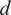 , and for each data segment, 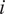 . Finally, the maximum excursion for each direction of each DOF is taken.
(16)

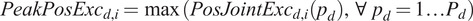



(17)

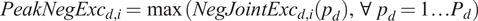

where 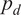 is a period of positive or negative phase, respectively, for DOF 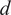 and 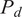 is the total number of periods of positive or negative phase, respectively, for DOF 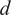 .

The phase for each DOF can also be stacked to describe the overall phase of each leg. For data segment 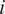 , a vector of phase vectors can be defined as follows:
(18)

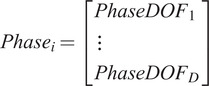

where 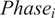 is a vector of phase vectors whose length is equal to the number of samples within the 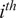 data segment.

#### Inter-joint coordination

The inter-joint coordination is defined as the pair-wise cross-correlations of joint angles of the hip, knee, and ankle joints of the predominant leg during full flexion and full extension. For each period of full flexion and full extension as defined in 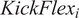 and 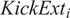 , the angular rate data are used to determine relative joint angles. The joint angles for the hip, knee, and ankle are considered 0 at the beginning of each period of full flexion and full extension. For each period of full flexion and full extension, the cross-correlation between each pair of joint angles is taken.

The average inter-joint coordination, 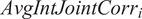 , is the mean cross-correlation between a joint pair during periods of full flexion and full extension.
(19)

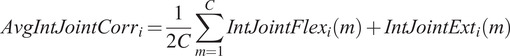

where 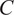 is the number of kick cycles within the 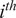 data segment, 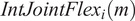 is a vector of cross-correlations between a joint pair during periods of full flexion, and 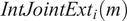 is a vector of cross-correlations between a joint pair during periods of full extension. In calculating 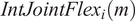 and 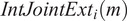 for the hip–ankle or knee–ankle pair, only periods of full flexion or full extension where the ankle is moving in phase with the hip and knee are considered. 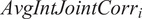 is calculated for the hip–knee, hip–ankle, and knee–ankle joint pairs.

### Determination of significant features

Spearman’s rho correlation coefficient is a non-parametric test that is used to measure the strength of association between the ranks of two variables, 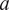 and 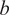 . Spearman’s rho is used here rather than Pearson’s correlation coefficient as it is a more robust measure of association and can measure the strength of nonlinear relationships. Values of this coefficient, 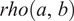 , range from −1 to +1, where a − 1 indicates a perfect negative monotonic correlation and a + 1 indicates a perfect positive monotonic correlation. A p-value, 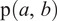 , is also determined for each 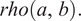 If 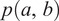 is less than 0.05, then 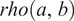 is considered significantly different from 0 at 95 % confidence.

A kinematic feature, 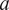 , is considered to be a significant predictor of age, 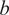 , if 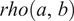 is considered significant. This coefficient is calculated using median values of the 1-min segments of normative data. Spearman’s rho determines whether or not the relationship between 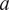 and 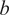 is significant, not the nature of the relationship. Therefore, a line of best fit is later used to determine whether the relationship between 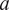 and 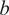 is linear or nonlinear.

## Normative kinematic features

Data from the 15 term infants, aged 1–8 months, is used in this study to determine normative kinematic features. Some infants were tested multiple times for a total of 31 sessions of data. Table 2 reports the ages tested and the number of 1-min data segments for each session of data. Ages are reported to the nearest half week. Data segments where the infant’s kicking frequency was greater than 0.9 and data segments where the infant’s frequency of rest was greater than 0.9 are considered outliers and excluded from analysis as this was considered a period of abnormal behavior. For each session of data, the median value of each kinematic feature is taken across all data segments. Spearman’s rho is then determined using these median values. Individual sessions are considered independent due to the extended period and significant growth between each session.

**Table 2.** Session information for term infant data

Infant ID	Sex	Gestational age (weeks)	Age tested (weeks)	Number of data segments
B	F	38	12	11
			18	18
D	F	39	8.5	7
			22	16
			36	12
E	F	39	8	14
			15	7
H	F	39	8	16
			22	13
			37	8
J	M	39.5	10	11
			18.5	13
			20.5	18
K	F	37.5	9	18
			16.5	13
			28.5	15
L	M	37	10.5	14
			19.5	15
			28.5	15
M	M	39.5	14	14
			24	15
O	M	38.5	13	15
P	M	40	12.5	13
			24.5	18
Q	F	39	6	16
R	F	40	12	9
S	F	40	13	9
T	M	40	13	14
			19.5	13
U	F	40	28	13

Results for significant features are reported in Tables 3 and 4. In Table 3, Spearman’s coefficients and associated p-values are reported for frequency, duration, acceleration, and inter-joint coordination metrics. Values associated with maximum joint excursion metrics are reported in Table 4. Coefficients associated with significant features are indicated in bold in these tables.

**Table 3.** Correlation coefficients for frequency, duration, acceleration, and inter-joint coordination metrics^a^

Group	Metric	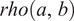	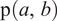
Frequencies (freq.)	Kicking	**−0.132**	< .01
	Bilateral	0.047	.343
	Predominant unilateral	**−0.353**	< .01
Durations (dur.)	Avg. kicking	**0.156**	< .01
	Avg. rest	**0.279**	< .01
	Max kicking	**−0.113**	.023
	Max rest	**0.214**	< .01
Acceleration (accel.)	Avg. kicking	**0.555**	< .01
	Peak kicking	**0.507**	< .01
Inter-joint coordination	Hip–knee	**0.219**	< .01
	Hip–ankle (in phase)	0.149	.073
	Knee–ankle (in phase)	**−0.121**	.016

a Correlation coefficients for significant features indicated in bold

**Table 4.** Correlation coefficients for max joint excursions of the predominant leg^a^

DOF	Direction	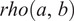	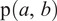
Hip flexion	Flexion	0.079	.115
	Extension	**−0.122**	.014
Hip abduction	Adduction	**0.153**	< .01
	Abduction	**−0.151**	< .01
Hip rotation	Internal rot.	**0.200**	< .01
	External rot.	**−0.232**	< .01
Knee flexion	Flexion	0.054	.283
	Extension	0.005	.917
Ankle flexion	Flexion	**0.432**	< .01
	Extension	**−0.485**	< .01
Ankle inversion)	Inversion	**0.328**	< .01
	Eversion	**−0.324**	< .01

a Correlation coefficients for significant features indicated in bold

### Significant kinematic features for term infants

Table 3 reports Spearman’s coefficients and associated p-values for frequency, duration, acceleration, and inter-joint coordination metrics. Coefficients for significant features are indicated in bold. The p-values for Spearman’s correlation coefficient show significant predictors of age for all frequency metrics except for Bilateral Frequency. Additionally, Spearman’s coefficient is significant for all duration and acceleration metrics. Finally, Spearman’s coefficient is considered significant for the hip–knee and knee–ankle inter-joint coordination, but not for the hip–ankle inter-joint coordination.

Table 4 reports Spearman’s coefficients and associated p-values for the maximum joint excursion metrics of the predominant leg. While Spearman’s coefficient is significant for both directions of hip abduction and hip rotation, Spearman’s coefficient is only significant for the extension direction of the hip flexion axis. Neither direction of knee flexion has a significant coefficient. Finally, coefficients for both directions for ankle flexion and ankle inversion are significant.

### Discussion of results

**Figure A1.**
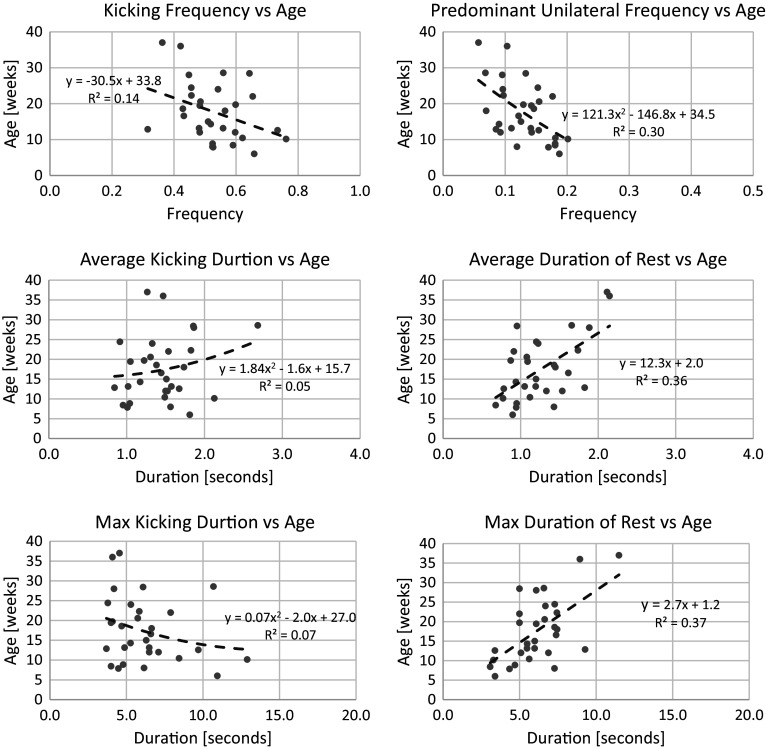
Lines of best fit representing normative trends for frequency and duration metrics.

**Figure A2.**
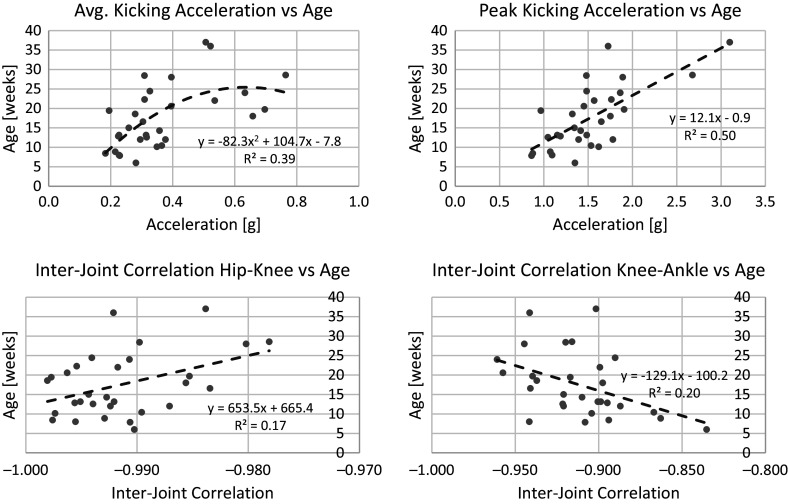
Lines of best fit representing normative trends for acceleration and inter-joint coordination metrics.

**Figure A3.**
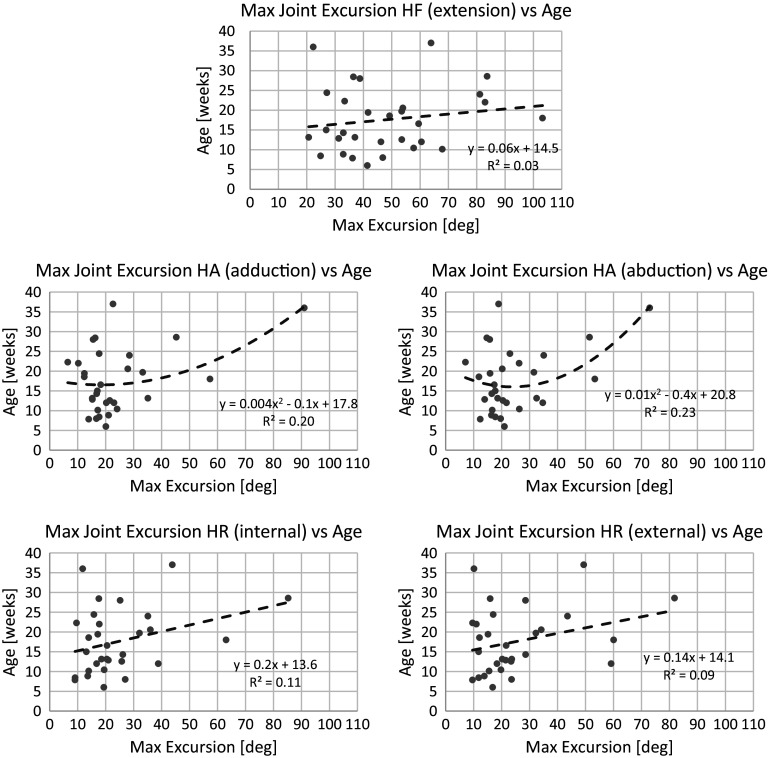
Lines of best fit representing normative trends for maximum joint excursion metrics for significant DOFs of the hip joint.

**Figure A4.**
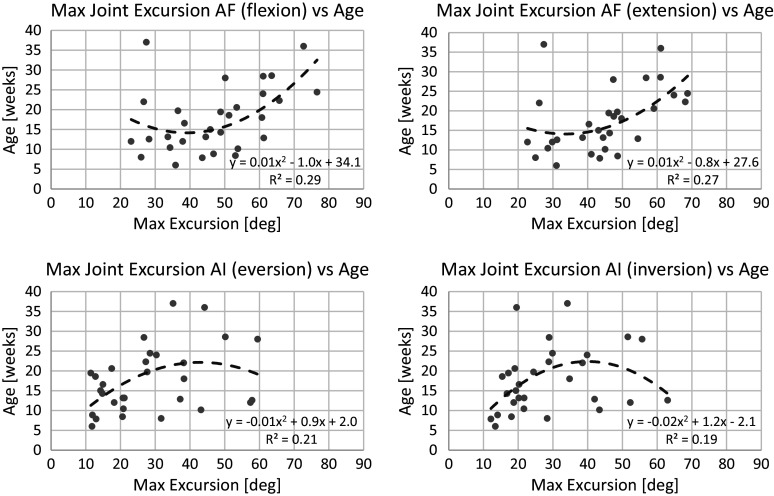
Lines of best fit representing normative trends for maximum joint excursion metrics for significant DOFs of the ankle joint.

Using the results from the correlation analysis shown in Tables 3 and 4, a line of best fit can be determined for significant kinematic features to create a model to predict infant age. Additionally, an 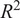 value is used to determine model fit. 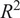 is a statistical measure that represents the proportion of the variance for a dependent variable that is explained by an independent variable in a regression model. This differs from correlation which explains the strength of the relationship between an independent and dependent variable. 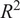 is determined as,
(21)

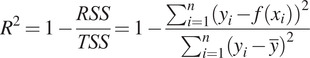

where 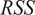 is the sum of squares of the residuals and 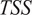 is the total sum of squares. Furthermore, 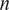 is the number of observations, 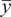 is the mean value of the dependent variable, and 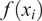 is the predicted value of 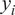 using the model. The line of best fit is determined from median values of each session of data. To avoid overfitting, either a linear or quadratic line of best fit is chosen. Plots of median values for term infants and models are shown in Figures A1–A4 in Appendix A. Reported for each model is the equation for the line of best fit and an 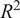 value.

Generally, term infants tend to kick less frequently, but for longer durations of time as they age. Term infants also tend to display longer periods of rest as they age. These trends in duration and frequency would indicate an infant’s movement becomes more deliberate as they age, displaying fewer periods of short spontaneous movement and more periods of sustained intentional movement. Additionally, values for acceleration and maximum joint excursion tend to increase as term infants age. Generally, as an infant ages, they are able to kick with more force and exhibit a larger range of motion. Finally, the inter-joint coordination between the knee and hip tends to decrease as an infant ages, indicating more decoupled movement of the joints.

These models represent normative values for each significant kinematic feature and can be used to provide a prediction of infant age. It is important to note that the predictions of each model should be analyzed individually as potential collinearities and dependencies between kinematic features have not been taken into account. This analysis will be included in future work when a comprehensive model to predict infant age will be created.

## Analysis of PT data

Data from the eight PT infants, aged 1–10 months, included in our study are compared to normative kinematic features. Some infants are tested multiple times for a total of 18 sessions of data. Table 5 reports the gestational age, ages tested, the adjusted age, and the number of 1-min data segments for each session of data. Ages are reported to the nearest half week. The adjusted age is calculated by subtracting the number of weeks the infant was born early from the infant’s birth age. A PT infant is considered motor developmentally delayed if the kinematic features associated with their spontaneous kicking are indicative of a younger infant, that is, a PT infant would display characteristics that are typical of a younger infant.

**Table 5.** Session information for preterm infant data

Infant ID	Sex	Gestational age (weeks)	Age tested (weeks)	Adjusted age (weeks)	Number of data segments
C_1_	F	32	20	12	13
C_2_	M	32	20	12	3
F	F	34	7.5	1.5	4
			12.5	6.5	12
			22.5	16.5	15
			30.5	24.5	12
			35.5	28.5	7
G1	M	33.5	8.5	2	12
G2	M	33.5	8.5	2	18
I	M	37	5	2	16
			10	7	11
			22.5	19.5	15
			28.5	25.5	18
N	F	36	14.5	10.5	15
			20.5	16.5	20
V	F	28	26	14	15
			35	23	16
			41	29	15

### PT kinematic features against normative values

Using the significant kinematic features identified in section “Normative kinematic features”, we develop normative models to predict infant age. Predictions of a PT infant’s age are determined by each normative model for all 1-min segments of kicking data. The median estimate for each session of data is recorded in Tables 6 and 7. If a model’s estimate is less than the PT infant’s age, the infant would be considered potentially delayed for the associated kinematic feature. Potential delays are indicated in Tables 6 and 7 with bold text.

**Table 6.** Median estimate of infant age from normative models (frequency, duration, and acceleration measures)

Infant ID	Age tested (weeks)	Kick freq.	Pred. unilateral freq.	Avg. kick duration	Avg. duration of rest	Max kick duration	Max duration of rest	Avg. accel.	Peak accel.
C_1_	20	**13.9**	**14.4**	**18.5**	**13.3**	**14.9**	**13.7**	**13.7**	**11.8**
C_2_	20	**14.0**	**15.9**	**18.5**	**17.1**	**16.8**	**18.3**	20.5	**19.6**
F	7.5	9.2	12.2	24.2	10.4	14.6	10.8	19.6	17.7
	12.5	16.6	14.3	18.4	13.2	16.4	16.2	16.9	15.6
	22.5	**14.3**	**15.0**	**19.2**	**14.5**	**17.9**	**13.0**	**21.9**	**13.8**
	30.5	**17.2**	**11.9**	**18.3**	**16.5**	**15.4**	**16.9**	**20.6**	**17.1**
	35.5	**15.3**	**17.6**	**18.4**	**15.3**	**19.4**	**13.3**	**21.9**	**20.4**
G1	8.5	23.4	20.4	15.7	18.8	22.1	24.5	9.7	10.4
G2	8.5	23.4	20.9	15.9	21.8	19.6	24.6	13.7	10.2
I	5	19.6	13.7	16.7	18.1	18.8	17.4	17.9	11.9
	10	20.4	**8.3**	17.7	23.2	19.4	18.1	16.0	10.7
	22.5	**20.0**	**18.5**	**17.5**	**19.4**	**19.0**	**19.2**	**20.2**	**15.8**
	28.5	**20.3**	**17.6**	**16.4**	**23.9**	**18.4**	**23.8**	**18.5**	**15.7**
N	14.5	16.7	20.1	19.2	18.5	16.1	**13.8**	17.0	19.5
	20.5	**19.8**	**16.7**	**16.0**	**15.8**	**18.6**	**18.2**	**16.5**	**14.8**
V	26	**18.7**	**11.5**	**16.0**	**13.6**	**19.8**	**9.5**	**5.4**	**7.7**
	35	**26.7**	**26.1**	**16.6**	46.7	**21.0**	**30.2**	**9.7**	**14.7**
	41	**17.7**	**23.1**	**20.6**	**27.3**	**18.0**	**19.4**	**23.6**	**32.3**

**Table 7.** Median estimate of infant age from normative models (inter-joint coordination (I-J C) and maximum joint excursion)

Infant ID	Age tested (weeks)	I-J C (hip–knee)	I-J C (knee–ankle)	Max joint excursion								
				HF (ext.)	HA (add.)	HA (abd.)	HR (int. rot.)	HR (ext. rot.)	AF (flex.)	AF (ext.)	AI (inv.)	AI (ever.)
C_1_	20	**18.4**	21.5	**18.0**	**16.8**	**16.4**	**16.1**	**16.6**	**14.9**	**15.3**	20.7	21.4
C_2_	20	21.7	**14.4**	**17.5**	**16.5**	**16.6**	**17.7**	**17.6**	**14.4**	20.2	**19.1**	21.6
F	7.5	19.9	15.1	19.5	17.1	17.5	21.2	19.3	15.6	14.6	22.0	22.1
	12.5	16.0	17.0	18.5	16.8	16.3	17.4	16.6	20.7	19.9	16.5	19.7
	22.5	**16.9**	**16.2**	**17.4**	**16.8**	**16.8**	**20.1**	**18.5**	**14.9**	**16.0**	**18.1**	**16.7**
	30.5	**15.9**	**10.6**	**17.9**	**16.7**	**16.6**	**16.4**	**16.5**	**15.8**	**18.5**	**20.6**	**19.6**
	35.5	**15.0**	**21.8**	**16.3**	**16.6**	**16.6**	**20.0**	**19.6**	**23.4**	**17.4**	**15.1**	**18.9**
G1	8.5	15.2	16.7	16.6	16.7	16.6	16.2	16.8	15.0	15.0	11.5	9.0
G2	8.5	16.9	18.2	16.9	16.9	16.3	17.7	17.6	17.2	16.8	**8.2**	**5.8**
I	5	16.8	23.5	15.9	16.8	16.2	16.7	16.4	15.1	15.0	12.1	14.5
	10	15.4	23.7	16.1	16.6	16.2	15.7	15.8	16.5	15.6	11.7	**9.3**
	22.5	**17.5**	**17.6**	**17.3**	**17.0**	**17.3**	**16.5**	**16.6**	**18.8**	**19.5**	**18.7**	**18.0**
	28.5	**14.1**	**6.1**	**17.2**	**17.0**	**18.2**	**16.7**	**16.7**	**16.7**	**18.7**	**13.9**	**16.1**
N	14.5	17.2	17.9	17.7	18.5	16.5	17.1	17.5	16.8	19.3	16.5	15.9
	20.5	**16.6**	**18.9**	**16.7**	**16.6**	**16.7**	**16.8**	**16.9**	**15.2**	**15.4**	**16.6**	**15.6**
V	26	**14.1**	**23.5**	**15.6**	**17.3**	**18.8**	**15.6**	**15.4**	**18.7**	**15.3**	**10.4**	**10.8**
	35	**16.5**	**26.5**	**15.5**	**17.0**	**18.0**	**17.3**	**17.8**	**18.5**	**16.8**	**15.9**	**11.9**
	41	**26.1**	**19.7**	**17.4**	**17.8**	**18.0**	**17.7**	**17.7**	**14.5**	**14.8**	**19.7**	**17.2**

Generally, PT infants kicked less frequently than their term counterparts at younger ages. As such, when predicting infant age using frequency measures, the predicted age of PT infants was greater than their actual age. However, around approximately 20 weeks, PT infants kicked more frequently than their term counterparts. As such, the predicted age of PT infants was estimated as less than their actual age, indicating potential delay. Similarly, when using average and maximum durations of kicking and rest measures, the predicted ages for PT infants younger than 20 weeks are greater than their actual age. At younger ages, PT infants tend to display longer periods of duration and rest compared to their term counterparts. However, as PT infants approached the 20 week mark, their average values of duration and rest did not increase on trend with their term counterparts. As such, the predicted age was estimated as less than their actual age, indicating potential delay for these features.

Estimates of PT infant age from acceleration and inter-joint coordination measures generally overpredicted PT infant age before the 20 week mark. These estimates indicate elevated values for acceleration and decreased values for inter-joint coordination for PT infants at young ages. Past the 20 week mark, while estimates of age do increase, these models underpredict PT infant age, indicating potential delay. The estimates for maximum joint excursion tend to increase as PT infants age, indicating a larger range of motion with an increase in age as is observed in normative data. Again, PT infants younger than 20 weeks are not typically indicated as delayed though delays are indicated for 20 weeks and beyond.

### Discussion of results

Generally for younger ages (less than 10 weeks), PT infants display kinematic values which suggest more mature, developed kicking as compared to normative values at their age of testing. Past the 10 week mark, predictions of PT infant age tend to approach the infant’s actual age. Finally, around the 20 week mark, predictions of PT infant age tend to indicate delayed motor development. In short, PT infants seem to display more mature kicking behavior prior to 10 weeks before falling behind around 20 weeks. A similar finding is also observed in Fetters et al. (2004). These results indicate that PT infants display different developmental trends in their spontaneous kicking as compared to term infants.

For term infants, the 20 week mark is significant. Term infants tend to kick less frequently, but for longer durations of time as they age. This trend was observed in previous work (Fry-Hilderbrand et al., 2021) and is believed to indicate the transition between spontaneous, involuntary movement to more deliberate, voluntary movement at around 20 weeks of age for term infants. During this transition, infants begin to break up basic motor patterns to develop more difficult motor skills, transitioning to more complex movement patterns. For PT infants, this transition between spontaneous and voluntary movement likely occurs later in life. For a 12 week PT infant observed in Fry-Hilderbrand et al. (2021), this transition is observed at 40 weeks of age or 28 weeks adjusted age.

As such, although it is unclear why PT infants seem to display more mature kicking at young ages, a possible explanation for the delays observed around 20 weeks is simply due to the underdevelopment of the PT infant’s muscles and brain. Term infants simply have a longer time to develop while in the womb. Thus, while term infants generally have an established foundation from which to grow, PT infants are simultaneously catching up in their physical development and increasing their motor repertoire. This results in a higher rate of change for these kinematic metrics for term infants than for PT infants. Finally, potential underdevelopment of the PT brain could make the transition between spontaneous and voluntary movement at 20 weeks more difficult for PT infants as compared to their term counterparts, ultimately resulting in motor delay.

Furthermore, rather than evidence of more mature kicking prior to the 20 week mark, potential fatigue due to the underdevelopment of PT infants may have been observed. Term infants are able to kick more frequently in short bursts of activity and with short periods of rest in between periods of activity. Conversely, PT infants kick less frequently due to their underdevelopment but tend to kick in longer durations. However, they require longer periods of rest between periods of activity to recover. As such, rather than a period of more advanced kicking, developmental patterns displayed by PT infants prior to 20 weeks could indicate an additional developmental period. During this period, PT motor development may be more focused on building strength and endurance rather than building their motor repertoire. Further study is needed to fully understand the implications of the observed differences between term and PT infant spontaneous kicking during this period.

At this time, it is unclear when observed delays are problematic and in need of intervention therapy. The group of PT infants observed in this study are considered low-risk, healthy PT infants. Though these infants are indicated as delayed by our normative models, the observed delays ultimately resolved as these infants aged. As such, observed delays may be acceptable as a PT infant is expected to resolve the delay without intervention therapy. Further work, with higher-risk PT infants, is needed to determine when observed delays are expected and acceptable versus when they become problematic and in need of potential intervention therapy.

Finally, though normative trends were established using term infant data in this work, a large-scale investigation including more typical infants is needed to create a better normative kicking database. A larger set of normative data would allow for the creation of more accurate and representative models of typical kicking behaviors.

## Conclusion

Our research aims to improve the early detection of infant motor delay by providing an objective measure of infant motor development outside of the clinical setting. The baby SmartyPants system is an embedded sensor suit intended for use in the home setting. This system is designed to be used with minimal training so that parents will be able to confidently deploy the system in their homes and collect infant kinematic kicking data over long periods of time. Automated analysis of the gathered data will inform clinicians of potential motor delay.

At this time, the system is not intended to provide direct feedback to the parents but to instead enable the extended collection of infant kinematic kicking data for clinical assessment. Additionally, this system is not intended to replace clinical diagnosis of motor disability but rather aid clinicians in identifying infants at-risk for motor delay. Finally, while the system is suitable for relatively short data collection periods, it is currently unknown how often one needs to change the coin-cell batteries during extended data collection. Frequent battery replacement could impact the usability and reliability of the system. Parents may be unwilling to frequently replace batteries or may be unable to reliably monitor individual sensor battery life. As such, ongoing work is being conducted to replace these coin-cell batteries with an alternative, single power source.

In this work, we introduce formulations of kinematic features identified in the clinical space using kicking data gathered from inertial sensors placed on the infant’s lower limb segments. We then identify which of these features are significant predictors of infant age and establish normative values at various ages. Finally, we offer an analysis of PT infant data compared to normative values. Though an infant may be indicated as delayed in one or more individual features, further analysis is needed to quantify the overall maturity associated with an infant’s kicking movements. Additionally, further work is needed to determine when observed delays are expected and acceptable versus when they become problematic and in need of potential intervention therapy.

Additionally, though not presented in this work, the goal of this research is to create a model to provide an estimate of infant development. Correlations between significant features must first be taken to identify potential dependencies and prevent introducing collinearity in the regression analysis. A multiple regression model to estimate developmental age will be created using the significant features presented in this work. An infant would be considered developmentally delayed if the estimate from this model indicated a developmental age younger than the age of the infant. Following the detection of these delays, intervention therapy can be provided by clinicians as needed, resulting in better outcomes for PT infants and improving their overall quality of life.

## Data Availability

Anonymized data can be made available to interested researchers upon request by email to the corresponding author.
